# Supplementary material for: Cancer-Related Pain Management in Suitable Intrathecal Therapy Candidates: A Spanish Multidisciplinary Expert Consensus
Source: Curr Oncol. 2023 Aug 1;30(8):7303–14. doi: 10.3390/curroncol30080530 (PMC10453610; doi:10.3390/curroncol30080530)
Supplement: Supplementary file 1 [file curroncol-30-00530-s001.zip › curroncol-2482181-supplementary.pdf]

Table S1. Number of professionals participating from the different Spanish regions.

Spanish regions	% (n)	Total population*
Galicia	19.4% (13)	2,695,645
Comunidad de Madrid	19.4% (13)	6,751,251
Andalucía	14.9% (10)	8,472,407
Cataluña	10.4% (7)	7,763,362
Castilla y León	7.5% (5)	2,383,139
Comunitat Valenciana	7.5% (5)	5,058,138
Extremadura	7.5% (5)	1,059,501
Región de Murcia	4.5% (3)	1,518,486
País Vasco	4.5% (3)	2,213,993
Canarias	1.5% (1)	2,172,944
Cantabria	1.5% (1)	584,507
Castilla - La Mancha	1.5% (1)	2,049,562
La Rioja	0%	319,796
Comunidad Foral de Navarra	0%	661,537
Ceuta	0%	83,517
Melilla	0%	86,261
Aragón	0%	1,326,261
Principado de Asturias	0%	1,011,792
Illes Balears	0%	1,173,008

*as of 2021, available at the Spanish National Statistics Institute (INE), www.ine.es.
